# Cytochrome P450 2U1 Is a Novel Independent Prognostic Biomarker in Breast Cancer Patients

**DOI:** 10.3389/fonc.2020.01379

**Published:** 2020-08-05

**Authors:** Bin Luo, Chuang Chen, Xiaoyan Wu, Dandan Yan, Fangfang Chen, Xinxin Yu, Jingping Yuan

**Affiliations:** ^1^Department of Pathology, Renmin Hospital of Wuhan University, Wuhan, China; ^2^Department of Breast and Thyroid Surgery, Renmin Hospital of Wuhan University, Wuhan, China

**Keywords:** cytochrome P450 2U1, breast cancer, prognostic biomarker, disease-free survival, overall survival

## Abstract

**Background:** The susceptibility of breast cancer is largely affected by the metabolic capacity of breast tissue. This ability depends in part on the expression profile of cytochrome P450 (CYPs). CYPs are a superfamily of enzymes with related catalysis to endogenous and exogenous bioactive substances, including xenobiotic metabolism, drugs, and some endogenous substances metabolism which activate cells and stimulate cell signaling pathways, such as arachidonic acid metabolism, steroid metabolism, fatty acid metabolism. Interestingly, CYP was electively expressed in different tumors, and mediated the metabolic activation of multiple carcinogens and participated in the activation and deactivation of tumor therapeutic drugs. However, the biological action of cytochrome P450 2U1 (CYP2U1) in breast carcinoma is little understood so far.

**Methods:** To investigate the biological value of CYP2U1 in breast carcinoma, we performed immunohistochemical (IHC) analysis and survival analysis based on clinico-pathological data of breast cancer.

**Results:** IHC analysis showed that the abundance of CYP2U1 protein was inversely proportional to the state of estrogen receptor(ER) (*P* < 0.05), and the lower the degree of tumor differentiation, the higher the protein abundance (*P* < 0.001). Additionally, compared with luminal tumors, the CYP2U1 protein content was more abundant in triple negative breast cancer (*P* < 0.05). Importantly, survival analysis showed that higher CYP2U1 protein levels predicted poor 5-year overall survival rate (*P* < 0.01), 5-year disease-free survival rate (*P* < 0.05), and 5-year metastatic-free survival rate (*P* < 0.01) for the entire enrolled breast cancer patients.

**Conclusions:** CYP2U1 is generally closely related to the clinicopathological characteristics and is also an adverse prognostic factor for breast carcinoma patients, indicating that CYP2U1 is engaged in the malignant progression of breast carcinoma.

## Introduction

Breast carcinoma is the most generalized type of cancer among women. It is one of the prime causes of cancer-related deaths in women and poses a serious threat to public health worldwide (1). On the grounds of the expression of estrogen receptor (ER), progesterone receptor (PR), and human epidermal growth factor receptor 2 (HER2), breast carcinoma is roughly classified into four different molecular subtypes, containing luminal, HER2-enriched, basal-like, and normal-like type (2). Many efforts have been done in the past few decades to better treat these tumor types (3–5). Nevertheless, the exact molecular mechanism of this highly heterogeneous neoplasm is still unknown at present. Identifying the key molecules that promote and maintain the malignant transformation of tumors helps to comprehensively understand the biology of breast cancer and ultimately supports people to develop new drugs to target new potential targets and implements precise treatment for breast cancer patients (6, 7).

Cytochrome P450 (CYP) enzymes are a multi-gene family of inducible monooxygenases that play an important role in the oxidative metabolism of a great deal of xenobiotics and endogenous components with biological activity (8, 9). Currently, based on nucleic acid homology, the human CYP superfamily is categorized into 18 families, 43 subfamilies, and 57 subtypes with catalytic functions (10). Some CYPs, particularly the dominating xenobiotic metabolic forms of CYPs, have been well-displayed in previous research, but little is known about the biological function of several CYPs identified in recent years. In addition, a single CYP showed characteristic cell type and tissue-specific expression patterns. Certain CYPs have also been exhibited to be expressed in carcinoma. In particular, CYP1B1 was overexpressed in a series of tumors and was an useful biomarker to predict chemotherapy response (11–13). CYPs might be potential cellulate targets in tumor cells for novel therapeutical exploitation and for targeting of personalized treatment.

CYP2U1 showed several unique characteristics in 57 human CYPs. For example, it exists in nearly everything organisms with highly conservative sequences. CYP2U1 gene is situated in chromosome 4 and carries 544 amino acid sequences presented 39% homology with other CYP2 family members. And, its specific genetic organization has only five exons, which are mainly located in the thymus and brain, and the protein sequence involves an abnormally long N-terminal region after the transmembrane helix, which contains 8 proline residues, and an insert of about 20 amino acids in which contains 5 arginine residues (14). So far, there are a small quantity of substrates, consisting of fatty acids, N-arachidonic acid serotonin, and drugs (15). Its main function is to catalyze the ω and ω-1 hydroxylation of fatty acids, and it is of great significance in intracellular signaling pathways (14, 16). Interestingly, this cytochrome is selectively distributed in tissues and selectively expressed in tumors (17). However, although CYP2U1 mutations have been implicated in certain pathological conditions, for instance, CYP2U1 was highly expressed in colorectal and ovarian cancer, and was closely related to tumor grade (18, 19), its biological role in breast carcinoma remains to be inquired in depth. Here, we carried out immunohistochemistry analysis in tissue microarray (TMA) to test the discrepant expression of CYP2U1 in normal and cancerous tissue, and to investigate the relation between the CYP2U1 expression and tumor differentiation, the expression of hormone receptors and HER2, and molecular subtypes at the protein level. In addition, we utilized Kaplan-Meier and COX regression plotter platforms to investigate the effect of CYP2U1 on the clinical outcome of breast carcinoma patients.

## Materials and Methods

### Tissue Sample

Breast specimens were acquired from the Department of Pathology at Renmin Hospital of Wuhan University. Samples of breast cancer were procured from a batch of 219 female patients with invasive breast carcinoma who undergone malignant breast tumor surgical excision (segmental or radical mastectomy) from January 2002 to December 2007. Sixty patients with benign breast lesions were selected as controls who were surgically treated by either mammotome or lumpectomy. Areas of tumor or control to be sampled for tissue microarray were first identified and was stained with HE to affirm the histopathologic findings and the adequacy of tissue specimens by two professional breast pathologist. Complete follow-up data were available for all patients and ranged from 1 to 60 months. Clinicopathological data included age at surgery, time of diagnosis, tumor type, histological grade, stage of disease, estrogen and progesterone receptor status, HER-2 expression level, time to recurrence and/or death(whichever came first), tumor size at diagnosis, chemotherapeutic strategy, and lymph-node status. The histopathology of all the breast neoplasm was acquired in the light of pathological diagnostic criteria of WHO (20). The study was approved by the Ethics Committee of Renmin Hosptial of Wuhan University.

### Immunohistochemistry

Specific primary antibody against CYP2U1 (ab252975, Abcam, Cambridge, MA, USA) was used for IHC at a dilution of 1:50. Immunohistochemical staining was performed as per instructions of the manufacturer. Sections of the TMA were dewaxed and rehydrated through a chain of xylenes and alcohols. Following that, the sections were incubated in 3% H_2_O_2_ diluted in methanol for 30 min in order to close biologically active sites of endogenous peroxidases. Antigen retrieval was implemented with citrate buffer (pH 8.0) at 98°C for 20 min. The slides were then allowed to cool to room temperature. After that, slides were incubated with primary antibody for 60 min at room temperature, and then incubated with biotinylated secondary antibody employing the Dako LSAB2 System-HRP (K0672, Dako Cytomation, Carpinteria, CA, USA) for 30 min, followed by washing with buffer so as to remove uncombined antibody. 3,3′diaminobenzidine (DAB) was utilized as the chromogen away from light applied for 3 min. Finally, sections were further washed within water and slightly counterstained with hematoxylin for 2 min. Slides with PBS, superseding the specific primary antibody, acted as blank controls.

### Quantification Evaluation

All slides were examined employing Olympus light microscopic (Olympus Corporation) and the staining intensity of immunoreactivity was evaluated by two independent observers using a semi-quantitative immunoreactivity scoring (IRS) system that was previously described for the evaluation of CYPs expression in TMA (18, 19). The evaluating pathologists performed IHC scores without prior knowledge of clinical pathological information. In the current research, CYP2U1 expression was chiefly localized to the cytoplasm in tumor samples, while its protein was principally expressed in the cellular nucleus of non-tumor samples. In IRS system, the intensity of immunostaining in each core was classified as four levels: 0(absence of immunostaining), 1(weak immunoreactivity), 2(moderate immunoreactivity), and 3(strong immunoreactivity). The difference in the evaluation of the case was minimal and was resolved by simultaneous re-evaluation by two experienced observers. According to previous reports, up to 15% core loss was accepted (21, 22). On the basis of the previously described standard protocol, the intensity stained tumor cells were employed to quantify the protein level of CYP2U1. In terms of the final staining score, 1–2 was grouped to low CYP2U1 protein expression, while 3 was grouped to high CYP2U1 protein expression.

### Statistical Analysis

The Student's *t*-test was employed to assess the differences in groups. Data were demonstrated as the mean ± standard error of the mean. Survival analysis was executed employing SPSS 20.0 software version (IBM Corp. Released 2011. IBM SPSS Statistics for Windows, Version 20.0. Armonk, NY: IBM Corp). In order to further verify the prognostic values of this new indicator, univariate and multivariate Cox proportional hazard regression analysis were performed for CYP2U1 expression and all clinicopathological features (including age, histological grade, T stage, ER status, PR status, HER2 expression, lymph node metastasis, and TNM staging). Only significant variables (*P* < 0.05) from the univariate analyses were allowed into the multivariate analysis. A *P* < 0.05 was considered statistically significant.

## Results

### Clinico-Pathological Characteristics of Patients

The study cohort included 219 breast carcinoma patients treated with surgical excision. All patients' clinicopathologic characteristics are shown in Table 1. The age at diagnosis ranged from 27 to 77 years (median, 50 years). Among all patients, 95 (43.4%) were diagnosed as Luminal subtype, 38 (17.4%) as HER2-enriched subtype, and 58 (26.5%) as TNBC. And notably, 28 patients with ER–/PR+ were not counted in classification of subtypes of breast cancer. There were 56 cases of tumors with high pathological grade and 163 cases of tumors with low-moderate pathological grade. There were 13 patients (5.9%) at early stages (I) and 206 patients (94.1%) at middle-advanced stages (II and III). A total of 54.8% patients (*n* = 120) had lymph node metastasis and 45.2% patients (*n* = 99) had no metastasis.

**Table 1:** Clinico-pathological information of patients in tissue microarray.

**Variables**	**Patient number (n)**	**Percentage (%)**
Age (years)		
≤50	137	62.6
>50	82	37.4
Menopausal state		
Yes	94	42.9
No	125	57.1
T stage		
T1	28	12.8
T2	148	67.6
T3	43	19.6
Lymph node metastasis		
0	99	45.2
≥1	120	54.8
Pathological grade		
I	36	16.4
II	127	58
III	56	25.6
TNM stage		
I	13	5.9
II	140	64
III	66	30.1
ER status		
Negative	124	56.6
Positive	95	43.4
PR status		
Negative	124	56.6
Positive	95	43.4
HER-2 status		
Negative	162	74
Positive	57	26
Molecular subtypes		
Luminal	95	43.4
HER2-enriched	38	17.4
TNBC	58	26.5

*ER, estrogen receptor; PR, progesterone receptor; HER2, human epidermal growth factor receptor 2; TNBC, triple-negative breast cancer; TNM, tumor-node-metastasis*.

### Differential Expression of CYP2U1 in Normal Breast Tissue and Cancer Tissue

**Figure 1:**
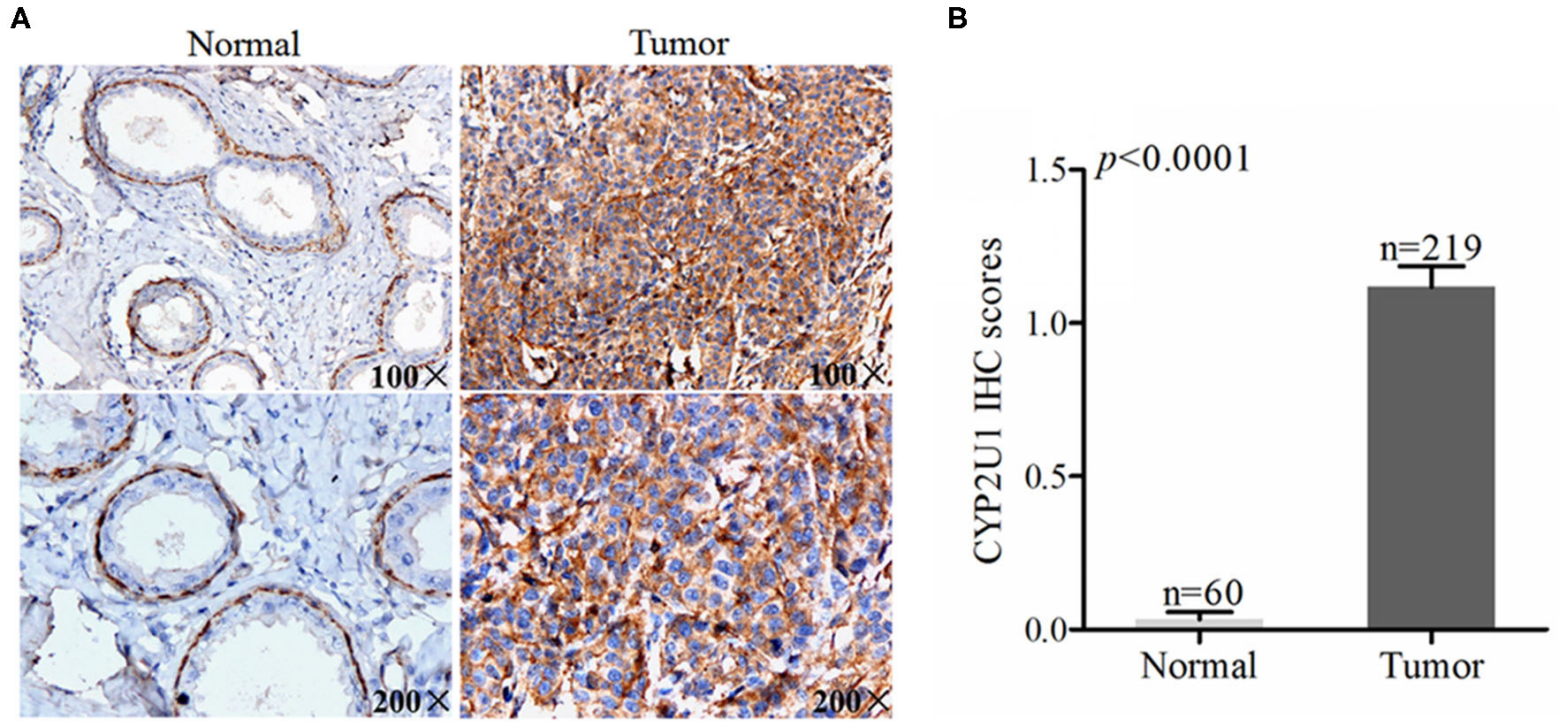
Analyses of cytoplasm CYP2U1 expression in normal breast and tumor tissues. **(A)** Representative images of CYP2U1 protein abundance in normal and breast tumortissues are shown. **(B)** Semi-quantitative result is displayed as the mean ± SEM.

To assess the protein levels of CYP2U1 in normal and cancerous specimens, we analyzed the TMA of 219 informative breast cancer patients via IHC. The results demonstrated that CYP2U1 was mainly detected in the cytoplasm of breast cancer tissue, while in non-cancer breast tissue, CYP2U1 was mainly located in the nucleus of cells derived from myoepithelial differentiation. The immunoreactivity for CYP2U1 in glandular epithelial-derived cells was negative in normal breast tissue. Typical IHC staining images of non-cancerous and cancerous tissues were exhibited in Figure 1A. Next, we inspected the potential relevance of CYP2U1 expression with breast carcinoma risk utilizing IHC staining scoring system. Our results showed that the protein level of CYP2U1 was distinctly higher in breast carcinoma tissues in comparison with normal tissues (Figure 1B, *P* < 0.0001).

### High Levels of CYP2U1 Were Connected With the Histopathological Grade of Breast Cancer

**Figure 2:**
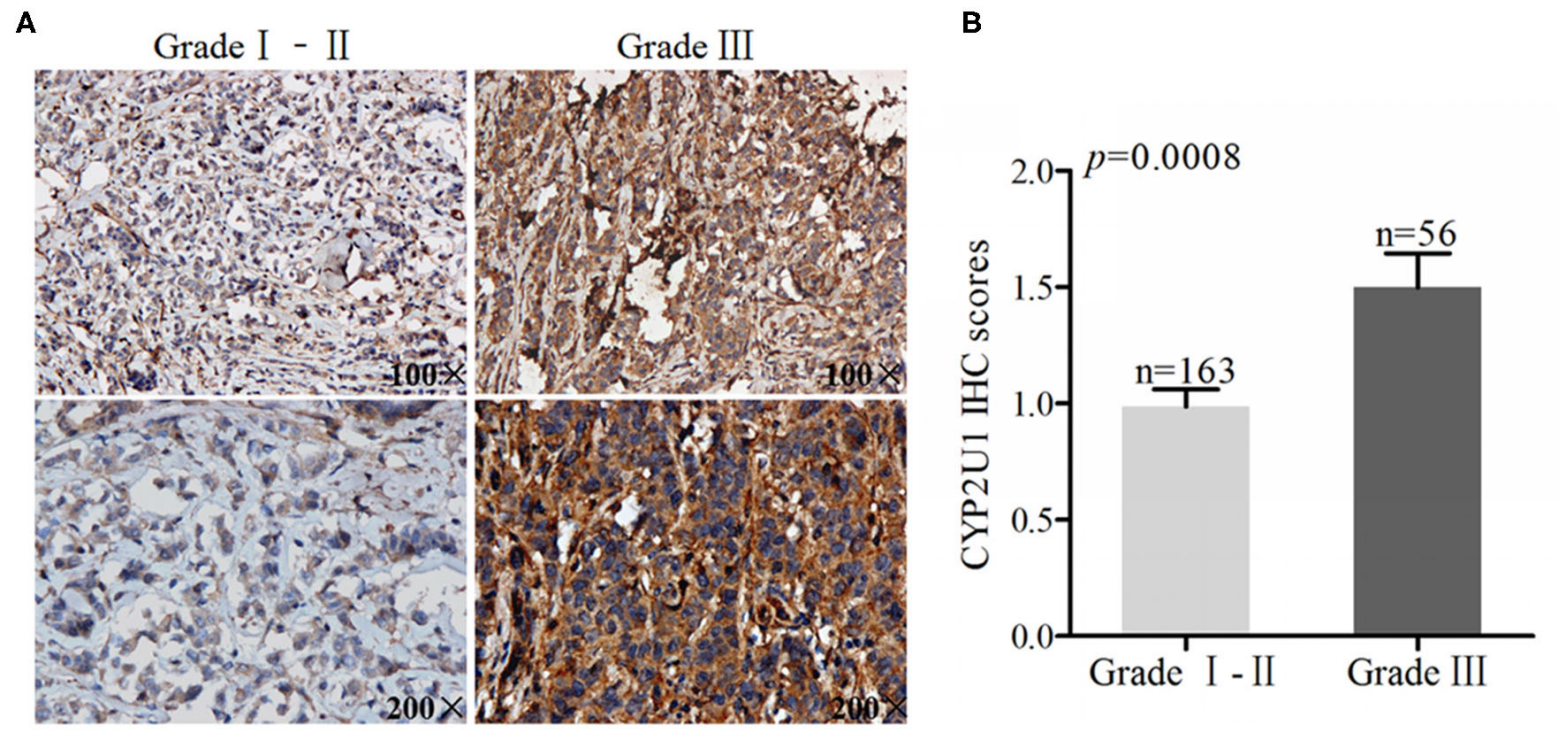
Analyses of cytoplasm CYP2U1 expression in low-grade and high-grade breast tumors. **(A)** Representative images of CYP2U1 protein abundance in low-grade and high-grade breast tumors are shown. **(B)** Semi-quantitative result is displayedas the mean ± SEM.

We also used the same method to evaluate the expression of CYP2U1 in breast cancer tissues with different degrees of differentiation. Typical IHC images of CYP2U1 in breast cancer tissues with different pathological grades were shown in Figure 2A. The IHC score results showed that the expression level of CYP2U1 in breast carcinoma specimen with pathological grade 3 is higher than that in breast cancer tissues with pathological grade 1–2 (*P* = 0.0008) (Figure 2B). It was proved that CYP2U1 expression level was negatively connected with the differentiation degree of breast tumors.

### Relevance Between CYP2U1 Expression and the Status of ER, PR, and HER2

**Figure 3:**
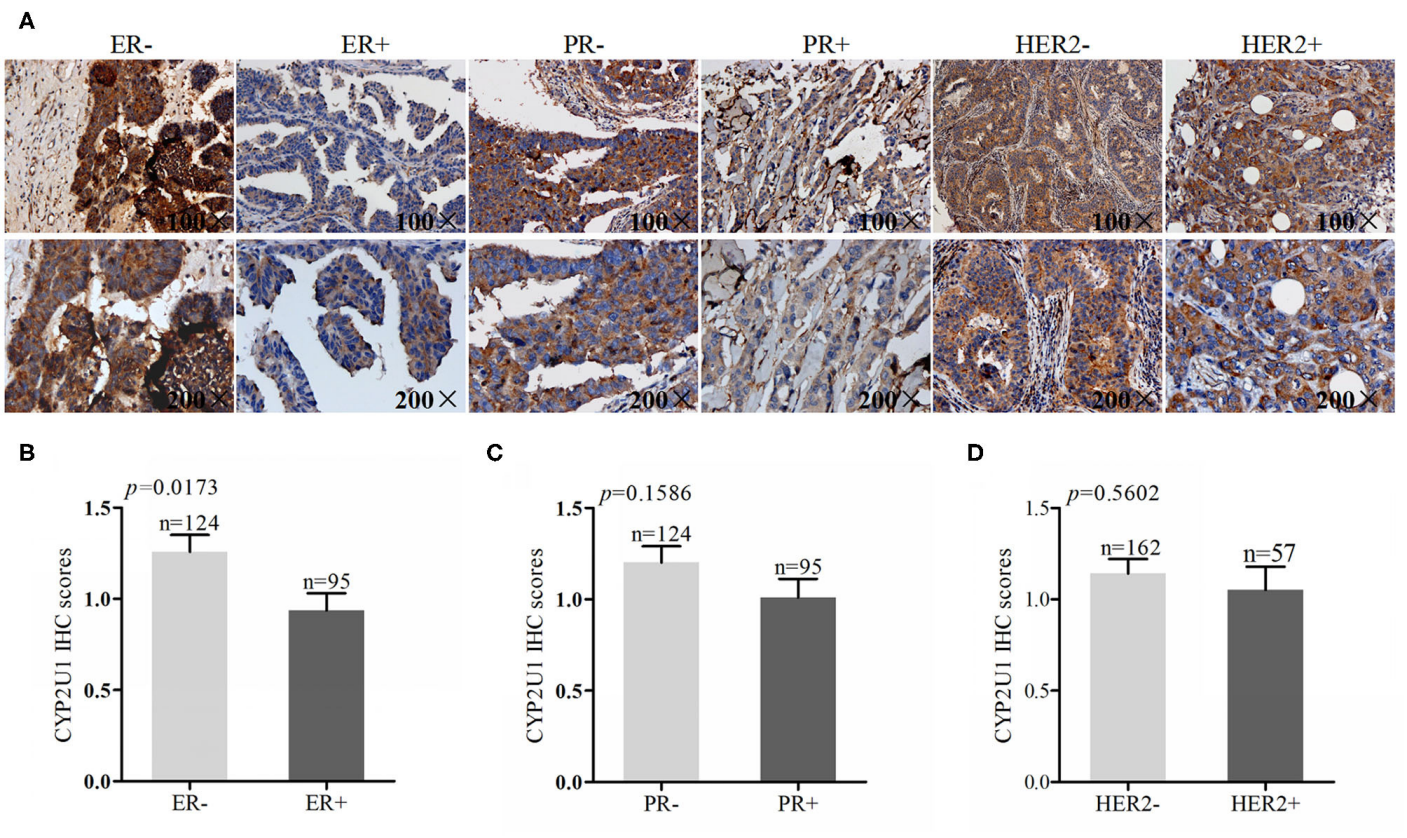
Analyses of CYP2U1 expression with status of ER, PR and HER2. **(A)** Representative images of CYP2U1 protein abundance in different status of ER, PR andHER2. **(B)** Semi-quantitative result of CYP2U1 protein abundance in ER-negative and ER-positive tumors tissue is displayed as the mean ± SEM. **(C)** Semi-quantitative result of CYP2U1 protein abundance in PR-negative and PR-positive tumors tissue is displayed as the mean ± SEM. **(D)** Semi-quantitative result about CYP2U1 protein abundance in HER2-negative and HER2-positive tumors tissue is displayed as the mean ± SEM.

To evaluate whether there was a correlation between CYP2U1 protein levels and the expression of hormone receptors and HER2, we combined IHC detection results with clinicopathologic data. IHC staining images of representative tissue specimens about negative/positive states of hormone receptors and HER2 were exhibited in Figure 3A, respectively. Next, IHC scores via employing semi-quantitative standard were measured as well. Research findings manifested that protein levels of CYP2U1 was dramatically higher in ER- in comparison with ER+ cancer tissues (*P* = 0.0173) (Figure 3B), while it was not reach statistical significance in the PR statue (*P* = 0.1586) (Figure 3C) and HER2 expression (*P* = 0.5602) (Figure 3D) of breast cancer patients. Thus, Statistical analysis on IHC score exposed that CYP2U1 expression was distinctly correlated with ER status.

### CYP2U1 Was Connected With Molecular Subtypes of Breast Carcinoma

**Figure 4:**
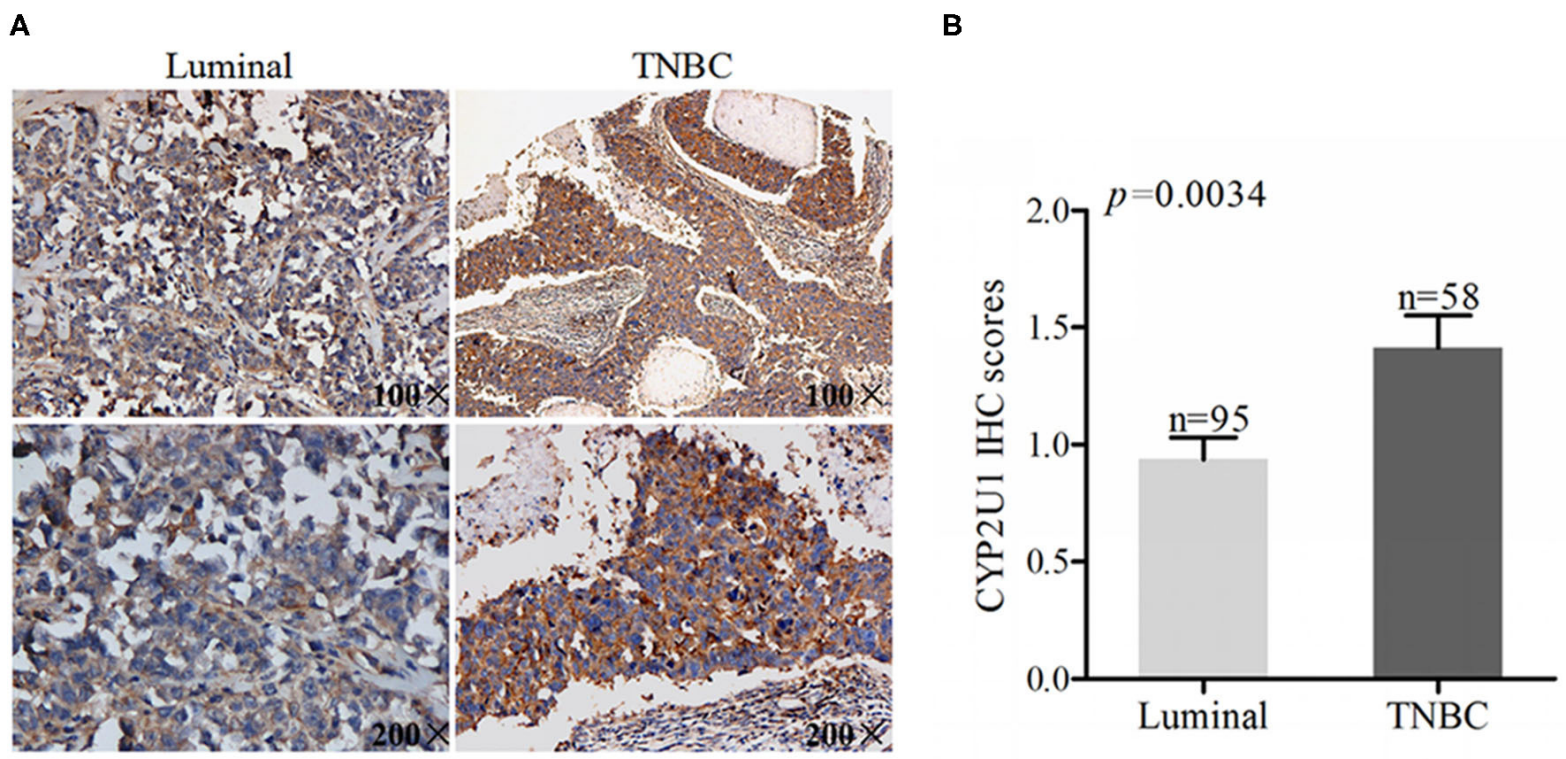
CYP2U1 is associated with molecular subtypes of breast cancer. **(A)** Representative images of IHC staining for luminal-type and TNBC breast cancer tissues were showed. **(B)** IHC scores revealed that higher level of CYP2U1 protein was significantly enhanced in TNBC tissues in comparison with luminal-type tissues, which was displayed as mean + SEM.

To expound the connection between CYP2U1 protein levels and the molecular subtypes of breast cancer, we examined the CYP2U1 cytoplasmic staining in 219 primary breast carcinoma. IHC staining images of representative tissue specimens for different molecular subtypes of breast cancer were exhibited in Figure 4A. The results of statistical analysis about staining scores exposed that CYP2U1 protein level was obviously elevated in TNBC tissues compared with luminal-type carcinoma specimens(*P* = 0.0034) (Figure 4B), while pairwise comparisons of other molecular types were not statistically significant. Collectively, we arrived at a conclusion that CYP2U1 was relatively abundant in highly malignant TNBC specimens compared with luminal-type specimens.

### High CYP2U1 Protein Predicted Poor Prognosis of Breast Cancer Patients

**Figure 5:**
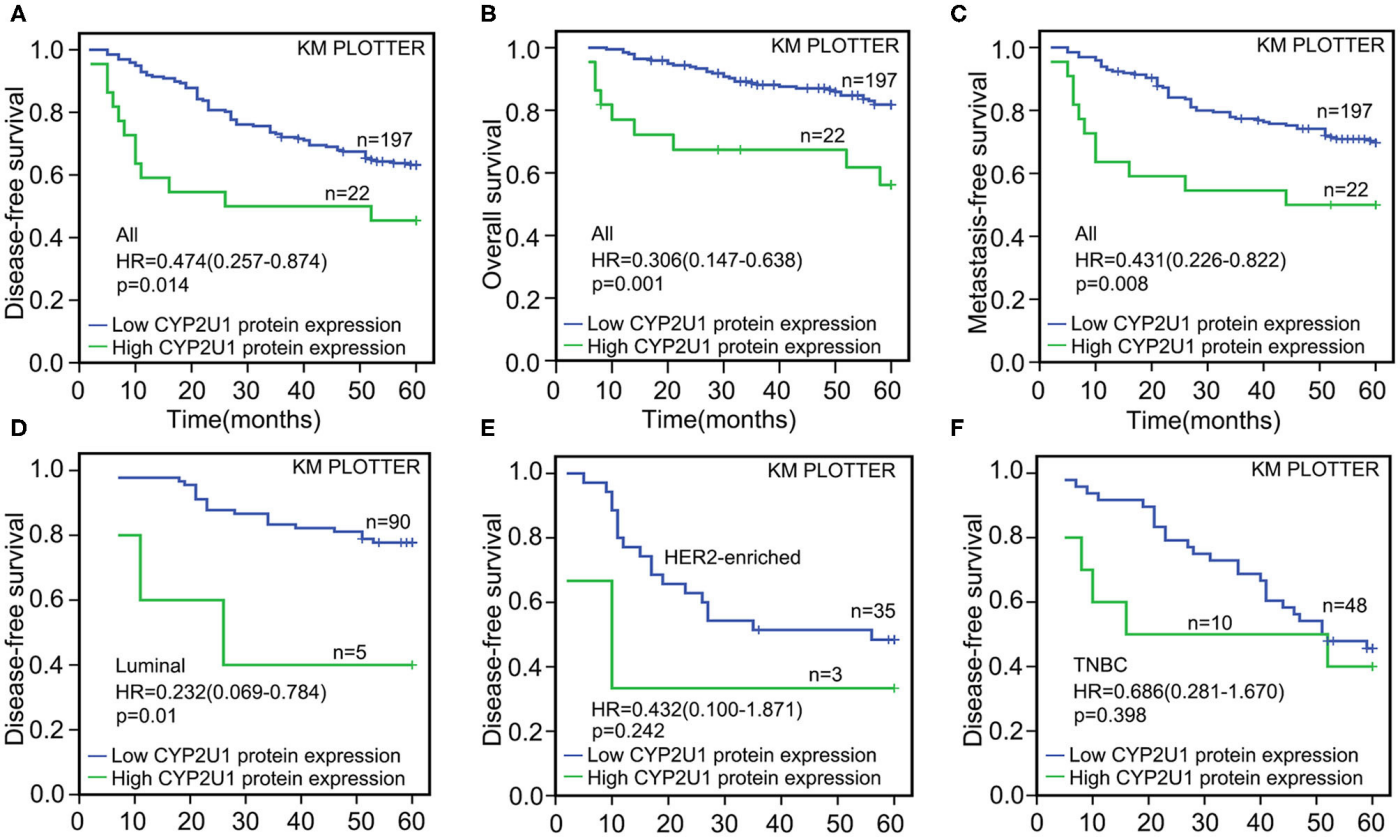
Kaplan-Meier survival curves for the correlation of CYP2U1 protein expression with breast cancer. Kaplan-Meier plotter analysis indicated that higher CYP2U1 protein expression was correlated with worse 5-DFS **(A)**, 5-OS **(B)**, and 5-MFS **(C)** at whole level, and patients with lower CYP2U1 protein level enjoyed longer time free from tumor recurrence and/or death among luminal subgroup **(D)**. Kaplan–Meier survival analysis of CYP2U1 protein expression was not statistically significant for 5-DFS in HER2 overexpression **(E)**, and TNBC **(F)** patients.

After 5 years of follow-up, the prognostic value of CYP2U1 in 219 breast carcinoma patients was determined. Hazard ratio (HR) and 95% confidence interval (CI) were applied to estimate the prognostic value of CYP2U1 protein levels in breast cancer patients. KM survival analysis and log-rank test disclosed statistically significant associations between higher levels of CYP2U1 protein and inferior rates of 5-year disease-free survival (5-DFS) (*P* = 0.014; HR = 0.474; 95% CI = 0.257–0.874), 5-year overall survival(5-OS) (*P* = 0.001; HR = 0.306; 95% CI = 0.147–0.638), and 5-year metastasis-free time(5-MFS) (*P* = 0.008; HR = 0.431; 95% CI = 0.226–0.822) (Figure 5). High expression level of CYP2U1 may be a new prognostic biomarker of breast cancer, and the clinical prognosis was poor. As shown in Tables 2, 3, univariate Cox proportional hazard regression analysis confirmed high histological grade, high T stage, low ER and PR, HER2 high-expression, late TNM stage, lymph node metastases and high expression of CYP2U1 were significant relevance in 5-DFS and 5-OS for breast cancer patients. These significant variables were then used in multivariate analysis. Similarly, it was further confirmed in multivariate analysis the high histological grade, high HER expression, lymph node metastasis and CYP2U1 high-expression was an important independent predictor of unfavorable 5-DFS and 5-OS.

**Table 2:** Univariate and multivariate Cox regression analysis of CYP2U1 protein expression for 5-year disease-free survival of breast carcinoma patients in tissues microarray.

**Characteristics**	**Univariate analysis**		**Multivariate analysis**	
	**HR (95% CI)**	***p*-value**	**HR (95% CI)**	***p*-value**
Age (≤50 vs. >50)	0.847 (0.548–1.310)	0.452	–	–
Pathological grade (I–II vs. III)	0.168 (0.109–0.260)	<0.0001^*^	0.261 (0.161–0.425)	<0.0001^*^
T stage (T1 vs. T2–T3)	0.210 (0.066–0.665)	0.003^*^	0.374 (0.116–1.200)	0.098
ER (negative vs. positive)	2.475 (1.531–4.001)	<0.0001^*^	1.334 (0.752–2.368)	0.325
PR (negative vs. positive)	1.714 (1.089–2.698)	0.018^*^	0.912 (0.534–1.557)	0.735
Her-2 (negative vs. positive)	0.481 (0.308–0.753)	0.001^*^	0.498 (0.308–0.803)	0.004^*^
Lymph node metastasis (0 vs. ≥1)	0.225 (0.132–0.384)	<0.0001^*^	0.272 (0.156–0.474)	<0.0001^*^
TNM stage (I vs. II–III)	0.044 (0.001–1.604)	0.009^*^	0	0.96
CYP2U1 protein expression (low vs. high)	0.474 (0.257–0.874)	0.014^*^	0.476 (0.247–0.917)	0.027^*^

*CI, confidence interval; HR, hazard ratio; ER, estrogen receptor; Her-2, human epidermal growth factor receptor-2; PR, progesterone receptor; TNM, tumor-node-metastasis*.

* *p < 0.05*.

**Table 3:** Univariate and multivariate Cox regression analysis of CYP2U1 protein expression for 5-year overall survival of breast carcinoma patients in the tissue microarray.

**Characteristics**	**Univariate analysis**		**Multivariate analysis**	
	**HR (95% CI)**	***p*-value**	**HR (95% CI)**	***p*-value**
Age (≤50 vs. >50)	0.656 (0.360–1.194)	0.164	–	–
Pathological grade (I–II vs. III)	0.131 (0.070–0.246)	<0.0001^*^	0.268 (0.136–0.527)	<0.0001^*^
T stage (T1 vs. T2–T3)	0.297 (0.072–1.227)	0.074	–	–
ER (negative vs. positive)	2.950 (1.453–5.991)	0.002^*^	1.432 (0.605–3.390)	0.415
PR (negative vs. positive)	2.165 (1.111–4.216)	0.02^*^	1.277 (0.576–2.828)	0.547
Her-2 (negative vs. positive)	0.317 (0.174–0.578)	<0.0001^*^	0.318 (0.165–0.614)	0.001^*^
Lymph node metastasis (0 vs. ≥1)	0.127 (0.050–0.322)	<0.0001^*^	0.144 (0.054–0.382)	<0.0001^*^
TNM stage (I vs. II–III)	0.045 (0.000–7.947)	0.072	–	–
CYP2U1 protein expression (low vs. high)	0.306 (0.147–0.638)	0.001^*^	0.259 (0.117–0.572)	0.001^*^

*CI, confidence interval; HR, hazard ratio; ER, estrogen receptor; Her-2, human epidermal growth factor receptor-2; PR, progesterone receptor; TNM, tumor node metastasis*.

* *p < 0.05*.

**Figure 6:**
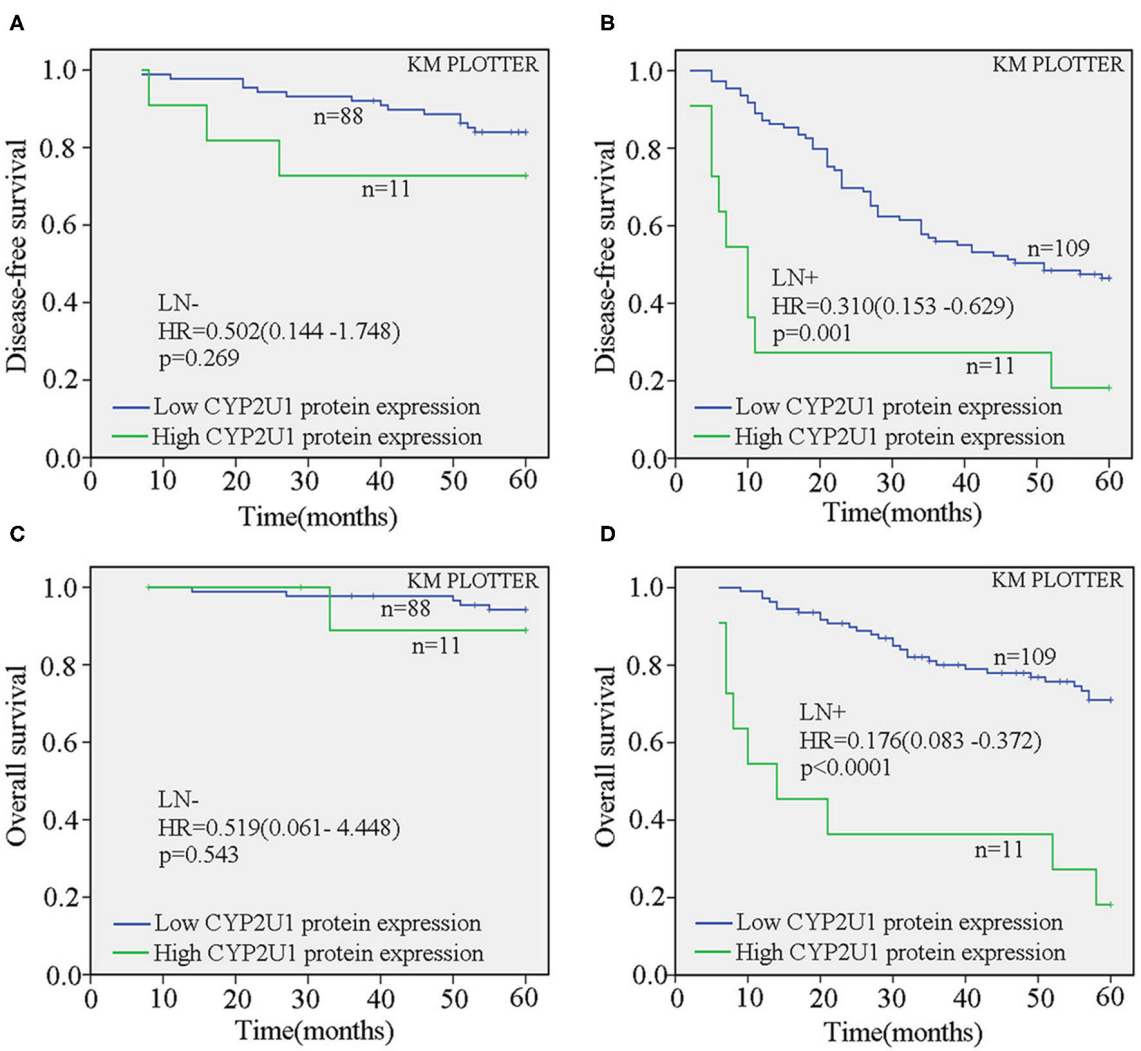
CYP2U1 expression was an unfavorable prognostic element for breast carcinoma patients with lymph node metastasis. Kaplan-Meier plotter analysis demonstrated that the relationship between CYP2U1 protein expression and 5-DFS **(A,B)** and 5-OS **(C,D)** of breast cancer patients with(or without) lymph node metastasis. LN–, lymph node metastasis negative; LN+, lymph node metastasis positive.

Next, we evaluated the correlation of CYP2U1 expression and the prognosis in disparate molecular subtypes of breast carcinoma. Kaplan–Meier survival analysis of CYP2U1 protein expression showed a significant relevance between high CYP2U1 expression and shorter 5-DFS in Luminal-type patients (*P* = 0.01; HR = 0.232; 95% CI = 0.069–0.784) (Figure 5D), with insufficient patients to assess 5-DFS in HER2-enriched and TNBC patients (Figures 5E,F). Moreover, Survival analysis of breast cancer patients with lymph node metastasis was implemented employing the KM Plotter platform to measure the clinical value of CYP2U1 in breast carcinoma. Research results indicated that patients with higher CYP2U1 protein expression in the LN+ group had worse 5-DFS [HR = 0.310 (0.153–0.629), *P* = 0.001; Figure 6B], and 5-OS [HR = 0.176 (0.083–0.372), *P* < 0.0001; Figure 6D], but no relevance was observe in the LN-group (Figures 6A,C).

## Discussion

CYP2U1 is well-known as a new type of human cytochrome in 2003. Among all CYPs, CYP2U1 is particularly fascinating because of its uniqueness in gene organization, protein sequence and tissue expression (14, 16). In previous studies, it was discovered that CYP2U1 mRNA is expressed at particularly high level in the thymus and cerebellum. As the further research continued, it was also found in the heart, bladder, prostate, uterus, testis, kidney and other tissues and organs (23). Recent studies have also found that the expression of CYP2U1 in colorectal and ovarian cancer tissues is higher than that of their adjacent paracancer tissues, and it is related to the malignancy of the tumor (18, 19). Consistent with previous studies, our study also indicated the protein content of CYP2U1 is prominently higher in breast cancerous tissues than in non-cancer tissues, and expression pattern of CYP2U1 had also changed from the original nuclear expression to cytoplasm expression. What is particularly noteworthy is that CYP2U1 is expressed only in myoepithelial nuclei of normal breast tissue. The presence or absence of myoepithelium is often used to determine whether there is infiltration of cancer cells. Using this feature, in pathological diagnosis, the IHC pattern of CYP2U1 expression may to some extent be served to distinguish cancer from non-cancer tissue in the future. The evidence needs to be further displayed that CYP2U1 molecule becomes an independent biomarker of myoepithelium.

The differentiation degree of tumor tissue can be divided into 3 levels: highly differentiated (histologic grade 1), moderately differentiated (histologic grade 2) and lowly differentiated (histologic grade 3). It has been reported that in colorectal and ovarian cancer, CYP2U1 was highly expressed in tumor cells with high pathological grade (18, 19). The same phenomenon has been observed in breast cancer. Our immunohistochemical analysis also showed that high CYP2U1 protein abundance was parallel to tissue grade and negatively correlated with the degree of differentiation. This provides more evidence to support that CYP2U1 is closely related to the grade of breast cancer and might have an adverse effect on the differentiation of breast carcinoma cells through some molecular mechanism. This is consistent with previous analysis implemented by Murray et al. (24) for 170 non-specific breast cancers, suggesting that breast tumors with high CYP2U1 protein level was often highly malignant.

Currently, ER, PR, and HER2 are important reference indicators for the drugs selection of breast cancer treatment, and also important indicators for prognostic evaluation (2). Therefore, we discussed whether there was any significant relevance between CYP2U1 protein level and the expression of hormone receptors and HER2. Our findings manifested that high content of CYP2U1 protein was detected in ER-negative breast cancer tissue, and there was an upward trend in the expression of CYP2U1 in PR-negative breast cancer tissue, although limited statistical value was observed based on the *t*-test. This finding is consistent with the report by Murray et al. (24) showing that CYP2U1 expression was obviously related to hormone receptors status. Thus, it can be seen that CYP2U1 expression is higher in breast cancer patients with hormone receptor negative.

As we all known, expression in ER,PR, and HER2 were employed to roughly divide breast carcinoma into four major molecular subtypes. Four subgroups exhibited diverse invasiveness of cancer cells and different treatment options. However, to date, almost no articles on the effects of molecular subtypes on CYP2U1 expression in humans have been published. In this study, using the tissue microarray and immuno-histochemistry technology, we for the first time revealed that the protein expression of CYP2U1 in TNBC is higher than that of Luminal subtype. More importantly, especially for TNBC patients with high CYP2U1 expression throughout breast tumors, the risk of tumor metastasis was high. In consistence, it was reported that the expression of CYP4A11 and CYP4A22 in TNBC tissues were higher than those in normal breast tissue. An increase of CYP4A/20-hydroxyeicosatetraenoic acid (20-HETE) activity was observed which promoted malignant transformation of breast ductal epithelium and accelerated cancer cell growth, thereby showing a high degree of biological malignant behavior in clinical manifestations (25). Borin et al. (26) also showed that the increased expression of CYP4A11 and CYP4A22 was associated with increased proliferation index of Ki-67 in TNBC tissues. The latest research confirmed that inhibiting the synthesis of 20-HETE led to the decreased migration and invasion of metastatic breast cancer cells (27). In this study, one possible explanation for high expression of CYP2U1 in TNBC tissues is that CYP2U1 are involved in the biosynthesis of 20-HETE. Therefore, in view of the obvious discrepancy in the expression of CYP2U1 in diverse molecular types of tumor specimens, the level of CYP2U1 may have a certain reference value in the selection of therapeutic regimen for breast cancer patients, and predicts that the patients with high CYP2U1 expression, have adverse clinical outcomes.

In some cancers, CYP2U1 acted as a prognostic predictor (28). In our research, we demonstrated high cytoplasmic expression of CYP2U1 is an independent prognostic biomarker of 5-DFS and 5-OS of breast cancer patients. Furthermore, high CYP2U1 expression was connected with several clinicopathological parameters consisting of TNM stage, tumor size, histopathologic grading, hormone receptors status, HER2 expression, and lymph node metastasis. CYP2U1 was first found in a large number of clinical data analysis to be related to the outcome of breast cancer patients. The fact that CYP2U1 protein was associated with 5-DFS and 5-OS was confirmed via conducting an independent IHC analysis based on 219 breast cancer patients. Several potential mechanisms behind the connection might be noticed. It was proved that CYP2U1 is a hydroxylase which metabolizes arachidonic acid and synthesizes 20-HETE, which is related to the proliferation of tumor cells, and is also upregulated in a variety of cancer (25). Moreover, arachidonicacid metabolic pathways accelerates tumor progression by activating dominating mitotic signaling pathways, including PI3K, MAPK, and Jun kinases (29–31). Lipid autacoid is a CYP-derived arachidonic acidmetabolite and may be participated in cancer development by regulating the survival and growth of cell-autonomous tumors or via regulating stromal setting, such as angiogenesis and inflammation which accelerates tumor progression (32). 20-HETE induced the transformation of tumor interstitial microenvironment to promote tumor phenotype, induced cell mitosis *in vivo* and *in vitro*, and promoted tumor angiogenesis, cell proliferation and metastasis (33–35). CYP synthesis inhibitors significantly shrunk the tumor size of intracranial gliomas and prolonged survival time in animal experiments (36). HET0016, an inhibitor of 20-HETE synthesis, reduced the level of proangiogenic factors and inhibited tumor growth of TNBC (26). Targeting 20-HETE synthesis also reduced the rate of pulmonary metastasis in an aggressive breast carcinoma model (27). Taken together, these researches suggested that there may be a link between increased CYP expression and cancer-promoting effects, which potentially explained the shorter 5-RFS, 5-OS, and 5-MFS observed in patients with high CYP2U1 expression.

However, it is undeniable that there are also some limitations in this study. Firstly, the prognostic value of CYP2U1 in breast cancer was discussed only at histological level. The exact role of CYP2U1 at the molecular level, especially for TNBC patients, still needs to be further explored in the follow-up research. Secondly, in this study, some patients received neoadjuvant therapy before surgery. So, what effect does neoadjuvant therapy have on the expression of CYP2U1 is not yet known, which needs to be assessed in the future study.

In conclusion, CYP2U1 was dramatically relevant to clinico-pathological characteristic of breast carcinoma, including histologic grade, T stage, the expression of hormone receptors, and HER2, lymph node metastasis and TNM stage, and it was enriched in TNBC tumors. Importantly, survival analysis of breast cancer data manifested that higher CYP2U1 protein expression forecasted poor prognosis among breast carcinoma population. Overall, CYP2U1 facilitated malignant biological behavior of breast carcinoma. We believe that these findings are important for further analysis of the molecular mechanism underlying the pro-tumor effects of CYP2U1 expression. Considering that CYP2U1 is involved in drug metabolism, future clinical trials should be need to detect the interaction of CYP2U1 and drugs in the context of adjuvant chemotherapy, especially for TNBC patients with high CYP2U1 expression. Identification of the possible interaction will provide more accurate treatment for breast cancer patients.

## Data Availability Statement

The raw data supporting the conclusions of this article will be made available by the authors, without undue reservation.

## Ethics Statement

The studies involving human participants were reviewed and approved by this study was approved by the Ethical Committee of Renmin Hospital of Wuhan University (WDRY2019-K010). The patients/participants provided their written informed consent to participate in this study. Written informed consent was obtained from the individual(s) for the publication of any potentially identifiable images or data included in this article.

## Author Contributions

JY designed and supervised the study. BL performed experiments and data analysis, drafted the manuscript, and prepared the figures. CC and XW collected the data of patients and followed up. XW and FC evaluated the immunohistochemical staining of all sections. DY and XY provided technical assistance with the data analysis. DY revised the manuscript. All authors have reviewed and approved this version of the manuscript.

## Conflict of Interest

The authors declare that the research was conducted in the absence of any commercial or financial relationships that could be construed as a potential conflict of interest.

## References

[1] DeSantis CE Ma J Gaudet MM Newman LA Miller KD Goding SA Breast cancer statistics, 2019. CA Cancer J Clin. (2019) 69:438–51. 10.3322/caac.21583 31577379

[2] Makki J. Diversity of breast carcinoma: histological subtypes and clinical relevance. Clin Med Insights Pathol. (2015) 8:23–31. 10.4137/CPath.S31563 26740749 PMC4689326

[3] Wang D Jiang W Zhu F Mao X Agrawal S. Modulation of the tumor microenvironment by intratumoral administration of IMO-2125, a novel TLR9 agonist, for cancer immunotherapy. Int J Oncol. (2018) 53:1193–203. 10.3892/ijo.2018.4456 29956749

[4] Ballinger TJ Meier JB Jansen VM. Current landscape of targeted therapies for hormone-receptor positive, HER2 negative metastatic breast cancer. Front Oncol. (2018) 8:308. 10.3389/fonc.2018.00308 30148117 PMC6095972

[5] Tong C Wu M Cho W To K. Recent advances in the treatment of breast cancer. Front Oncol. (2018) 8:227. 10.3389/fonc.2018.00227 29963498 PMC6010518

[6] Xu H Yu S Liu Q Yuan X Mani S Pestell RG. Recent advances of highly selective CDK4/6 inhibitors in breast cancer. J Hematol Oncol. (2017) 10:97. 10.1186/s13045-017-0467-2 28438180 PMC5404666

[7] Matsuhashi N Takahashi T Matsui S Tanahashi T Imai H Tanaka Y. A novel therapeutic strategy of personalized medicine based on anti-epidermal growth factor receptor monoclonal antibodies in patients with metastatic colorectal cancer. Int J Oncol. (2018) 52:1391–400. 10.3892/ijo.2018.4322 29568913 PMC5873832

[8] Guengerich FP. Intersection of the roles of cytochrome P450 enzymes with xenobiotic and endogenous substrates: relevance to toxicity and drug interactions. Chem Res Toxicol. (2017) 30:2–12. 10.1021/acs.chemrestox.6b00226 27472660 PMC5293730

[9] Elfaki I Mir R Almutairi FM Duhier F. Cytochrome P450: polymorphisms and roles in cancer, diabetes and atherosclerosis. Asian Pac J Cancer Prev. (2018) 19:2057–70. 10.22034/APJCP.2018.19.8.2057 30139042 PMC6171375

[10] Nelson DR Zeldin DC Hoffman SM Maltais LJ Wain HM Nebert DW. Comparison of cytochrome P450 (CYP) genes from the mouse and human genomes, including nomenclature recommendations for genes, pseudogenes and alternative-splice variants. Pharmacogenetics. (2004) 14:1–18. 10.1097/00008571-200401000-00001 15128046

[11] Chun YJ Kim D. Cancer activation and polymorphisms of human cytochrome P450 1B1. Toxicol Res. (2016) 32:89–93. 10.5487/TR.2016.32.2.089 27123158 PMC4843978

[12] Mitsui Y Chang I Fukuhara S Hiraki M Arichi N Yasumoto H. CYP1B1 promotes tumorigenesis via altered expression of CDC20 and DAPK1 genes in renal cell carcinoma. BMC Cancer. (2015) 15:942. 10.1186/s12885-015-1951-0 26626260 PMC4665921

[13] Su JM Lin P Wang CK Chang H. Overexpression of cytochrome P450 1B1 in advanced non-small cell lung cancer: a potential therapeutic target. Anticancer Res. (2009) 29:509–15. 19331196

[14] Karlgren M Backlund M Johansson I Oscarson M Ingelman-Sundberg M. Characterization and tissue distribution of a novel human cytochrome P450-CYP2U1. Biochem Biophys Res Commun. (2004) 315:679–85. 10.1016/j.bbrc.2004.01.110 14975754

[15] Siller M Goyal S Yoshimoto FK Xiao Y Wei S Guengerich FP. Oxidation of endogenous N-arachidonoylserotonin by human cytochrome P450 2U1. J Biol Chem. (2014) 289:10476–87. 10.1074/jbc.M114.550004 24563460 PMC4036169

[16] Chuang SS Helvig C Taimi M Ramshaw HA Collop AH Amad M. CYP2U1, a novel human thymus- and brain-specific cytochrome P450, catalyzes omega- and (omega-1)-hydroxylation of fatty acids. J Biol Chem. (2004) 279:6305–14. 10.1074/jbc.M311830200 14660610

[17] Dhers L Ducassou L Boucher JL Mansuy D. Cytochrome P450 2U1, a very peculiar member of the human P450s family. Cell Mol Life Sci. (2017) 74:1859–69. 10.1007/s00018-016-2443-3 28083596 PMC11107762

[18] Downie D McFadyen MC Rooney PH Cruickshank ME Parkin DE Miller ID. Profiling cytochrome P450 expression in ovarian cancer: identification of prognostic markers. Clin Cancer Res. (2005) 11:7369–75. 10.1158/1078-0432.CCR-05-0466 16243809

[19] Kumarakulasingham M Rooney PH Dundas SR Telfer C Melvin WT Curran S. Cytochrome p450 profile of colorectal cancer: identification of markers of prognosis. Clin Cancer Res. (2005) 11:3758–65. 10.1158/1078-0432.CCR-04-1848 15897573

[20] Lakhani SR Ellis IO Schnitt S Tan PH van de Vijver MJ WHO Classifciation of tumours of the breast. In: Lokuhetty D Watanabe R editors. WHO Classifcation of Tumours. 4th ed Lyon: IARC Press (2012). p. 82–134.

[21] Dundas SR Lawrie LC Rooney PH Murray GI. Mortalin is over-expressed by colorectal adenocarcinomas and correlates with poor survival. J Pathol. (2005) 205:74–81. 10.1002/path.1672 15532096

[22] Dhir R. Tissue microarrays: an overview. Methods Mol Biol. (2008) 441:91–103. 10.1007/978-1-60327-047-2_6 18370313

[23] Choudhary D Jansson I Stoilov I Sarfarazi M Schenkman JB. Expression patterns of mouse and human CYP orthologs (families 1–4) during development and in different adult tissues. Arch Biochem Biophys. (2005) 436:50–61. 10.1016/j.abb.2005.02.001 15752708

[24] Murray GI Patimalla S Stewart KN Miller ID Heys SD. Profiling the expression of cytochrome P450 in breast cancer. Histopathology. (2010) 57:202–11. 10.1111/j.1365-2559.2010.03606.x 20716162

[25] Alexanian A Miller B Roman RJ Sorokin A. 20-HETE-producing enzymes are up-regulated in human cancers. Cancer Genomics Proteomics. (2012) 9:163–9. 22798501 PMC3601443

[26] Borin TF Zuccari DA Jardim-Perassi BV Ferreira LC Iskander AS Varma NR. HET0016, a selective inhibitor of 20-HETE synthesis, decreases pro-angiogenic factors and inhibits growth of triple negative breast cancer in mice. PLoS ONE. (2014) 9:e116247. 10.1371/journal.pone.0116247 25549350 PMC4280215

[27] Borin TF Shankar A Angara K Rashid MH Jain M Iskander A. HET0016 decreases lung metastasis from breast cancer in immune-competent mouse model. PLoS ONE. (2017) 12:e0178830. 10.1371/journal.pone.0178830 28609459 PMC5469456

[28] Biswas NK Das S Maitra A Sarin R Majumder PP. Somatic mutations in arachidonic acid metabolism pathway genes enhance oral cancer post-treatment disease-free survival. Nat Commun. (2014) 5:5835. 10.1038/ncomms6835 25517499

[29] Hyde CA Missailidis S. Inhibition of arachidonic acid metabolism and its implication on cell proliferation and tumour-angiogenesis. Int Immunopharmacol. (2009) 9:701–15. 10.1016/j.intimp.2009.02.003 19239926

[30] Hii CS Moghadammi N Dunbar A Ferrante A. Activation of the phosphatidylinositol 3-kinase-Akt/protein kinase B signaling pathway in arachidonic acid-stimulated human myeloid and endothelial cells: involvement of the ErbB receptor family. J Biol Chem. (2001) 276:27246–55. 10.1074/jbc.M103250200 11359783

[31] Paine E Palmantier R Akiyama SK Olden K Roberts JD. Arachidonic acid activates mitogen-activated protein (MAP) kinase-activated protein kinase 2 and mediates adhesion of a human breast carcinoma cell line to collagen type IV through a p38 MAP kinase-dependent pathway. J Biol Chem. (2000) 275:11284–90. 10.1074/jbc.275.15.11284 10753939

[32] Panigrahy D Kaipainen A Greene ER Huang S. Cytochrome P450-derived eicosanoids: the neglected pathway in cancer. Cancer Metastasis Rev. (2010) 29:723–35. 10.1007/s10555-010-9264-x 20941528 PMC2962793

[33] Borin TF Angara K Rashid MH Achyut BR Arbab AS. Arachidonic acid metabolite as a novel therapeutic target in breast cancer metastasis. Int J Mol Sci. (2017) 18:2661. 10.3390/ijms18122661 29292756 PMC5751263

[34] Alexanian A Sorokin A. Targeting 20-HETE producing enzymes in cancer - rationale, pharmacology, and clinical potential. Onco Targets Ther. (2013) 6:243–55. 10.2147/OTT.S31586 23569388 PMC3615879

[35] Chen L Ackerman R Saleh M Gotlinger KH Kessler M Mendelowitz LG. 20-HETE regulates the angiogenic functions of human endothelial progenitor cells and contributes to angiogenesis *in vivo*. J Pharmacol Exp Ther. (2014) 348:442–51. 10.1124/jpet.113.210120 24403517 PMC3935142

[36] Zagorac D Jakovcevic D Gebremedhin D Harder DR. Antiangiogenic effect of inhibitors of cytochrome P450 on rats with glioblastoma multiforme. J Cereb Blood Flow Metab. (2008) 28:1431–9. 10.1038/jcbfm.2008.31 18414496 PMC2637201

